# Transformation-optical Fan-beam Synthesis

**DOI:** 10.1038/srep20530

**Published:** 2016-02-05

**Authors:** Rui Yang, Xianghui Kong, Hui Wang, He Su, Zhenya Lei, Jing Wang, Aofang Zhang, Lei Chen

**Affiliations:** 1 School of Electronic Engineering. Xidian University, Xi’an 710071, Peoples R China

## Abstract

Gradient-index dielectric lenses are generated based on the coordinate transformation by compressing the homogeneous air spaces quasi-conformally towards and outwards the primary source. The three-dimensional modeling is then performed through revolving the prescribed transformational media 180 degrees around the focal point to reach the architecture of barrel-vaults. It is found that all these two- and three-dimensional transformation-optical designs are capable of producing fan-beams efficiently over a broad frequency range with their main lobes possessing the narrow beamwidth in one dimension and the wide beamwidth in the other, while having the great ability of the wide angular scanning. Finally, we propose to construct such four types of fan-beam lenses through multiple-layered dielectrics with non-uniformed perforations and experimentally demonstrate their excellent performances in the fan-beam synthesis.

With simultaneously high gain and wide azimuth coverage, fan-beam radiator has been widely adopted in various applications among different disciplines^1^. It demonstrates highly efficiency for the bone scan in the medical imaging^2^, yields a significant link-budget advantage for the indoor wireless communication^3^, and also presents superior qualities for the height-finding as well as the obstacle detection in the radar system^4^,^5^,^6^.

The truncated parabolic reflector (TPR) has been so far the most commonly used device to produce the fan-beams^7^. Due to the inequality of the radiating aperture, the primary spherical waves from the focus are collimated differently. The narrower beam-width is achieved in the dimension where the reflector has the intact radiating aperture and the wider beam-width is obtained in the other dimension. The dielectric lens, at the same time, has also been proved to be a promising candidate to synthesize the fan-beams. Different from the three-dimensional (3D) construction of the TPR, most of the lens designs only adopt two-dimensional (2D) structures, such as the cylindrical Luneburg lenses^8^,^9^,^10^, and the up-to-date meta-surface lenses^11^,^12^,^13^,^14^, serving in the parallel plate waveguides or on the grounded metamaterial surfaces. These demonstrations successfully fulfill the functionality as fan-beam generators to collimate the radiation in the lens locating plane and maintain the beam pattern nearly unchanged in others, possessing the merits of low profile and convenience in the conformal design. However, such 2D proposals of the thin sheets also constrain their application at the same time due to the structural limitation of small perception windows connecting the outside in the direction of the main beam. Therefore, the practical utilization of the dielectric lenses compared with the ubiquitous TPRs in the radar antenna system for the fan-beam applications is still limited.

Transformation optics^15^,^16^,^17^, as a promising strategy to control the electromagnetic fields since 2006, has been widely adopted to build up the new antenna system^18^,^19^,^20^,^21^,^22^,^23^,^24^. These proposals, either based on the 2D coordinate transformation to synthesize the flat lenses, or creating the 3D construction of the transformational media by rotating the 2D permittivity map around its symmetry axis^25^, have demonstrated the great efficiency to collimate the cylindrical wave from a primary line source or the spherical wave from an isotropic point source. Although 2D lenses should be considered as the valid candidate for fan-beam lenses, the existing literatures have scarcely mentioned the synthesis of fan-beams using the transformation optics, with most of the 2D designs focusing on the perfect generation of the directive radiations, and the 3D set-ups aiming for the sharp pencil-beams. Transformation optics, having much wider applications in the wave control, should be powerful for different kinds of beam-forming and function as a very useful tool for the design of fan-beams. Based on these considerations, we demonstrate the transformational constructions of the 2D and the 3D lenses for the fan-beam generation in this paper. The homogeneous air spaces are compressed quasi-conformally based on the 2D coordinate transformation to come up with two kinds of the gradient-index dielectric lenses having the plano-concave profile and flat profile. Different from the conventional 3D transformation-optical pencil-beam lenses, we carry out the 3D modeling through revolving the prescribed transformational media 180 degrees around the focal point to reach the architecture of barrel-vaults. We show that all these transformation-optical designs will produce fan-beams efficiently with their main lobes possessing the narrow beamwidth in one dimension and the wide beamwidth in the other. In addition, we continue to verify the functionality of these fan-beam generators over a broad frequency range and demonstrate their great abilities in the wide angular scanning. Finally, we propose to utilize the non-uniformed perforated F4B layers to construct both of the 2D and the 3D fan-beam lenses and also present their excellent performances in the fan-beam synthesis through the experimental verification.

## Results

**Figure 1:**
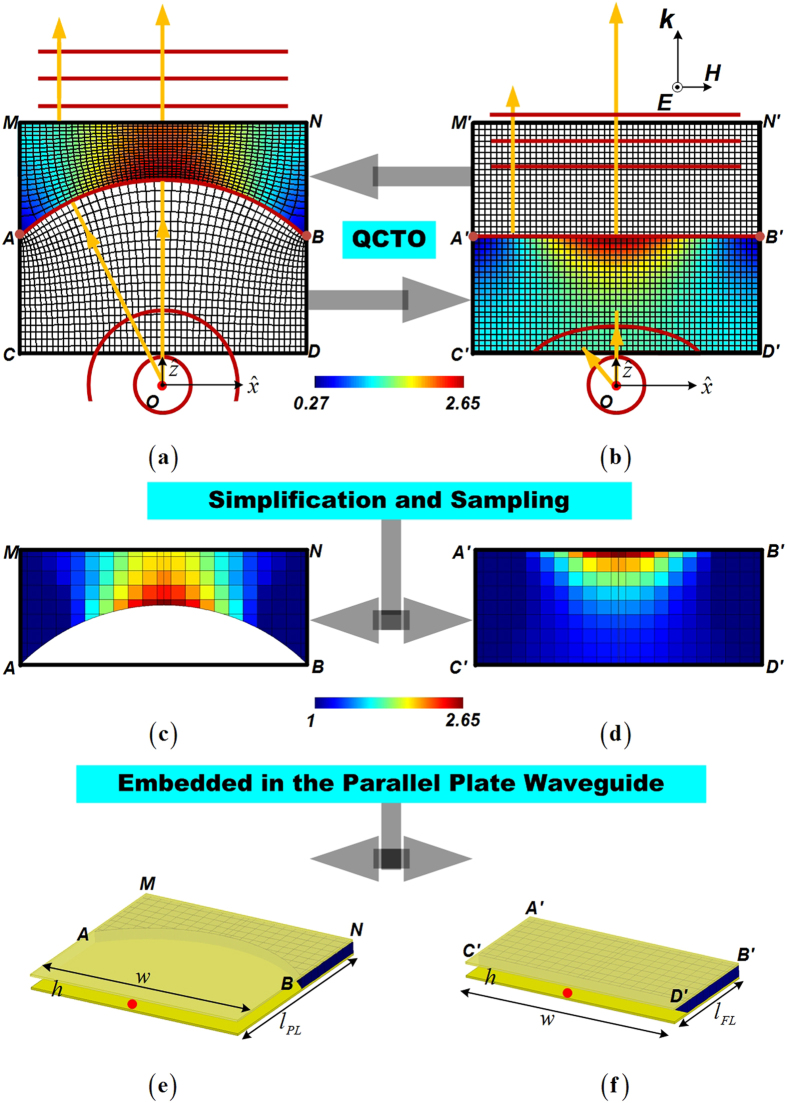
Design procedure of the 2D fan-beam lenses . The QCTO prescribed PL (**a**), and FL (**b**) with *l*_*AC*_ = *l*_*A’C’*_ = *l*_*AM*_ = *l*_*A’M’*_ = 80 mm, *l*_*CD*_ = *l*_*C’D’*_ = 200 mm, and the radius of the quadrant circle *AB* of 100√2 mm. In every transformational space of the *ACDB, A’C’D’B’, A’B’N’M’*, and *ABNM*, we have 50 × 20 quasi-orthogonal or orthogonal grids. The focal lengths of the PL and the FL are 100√2 mm and 20 mm, respectively. Simplified versions of the PL (**c**), and the FL (**d**). Schematic imaging of the PL (**e**), and the FL (**f**) embedded in the parallel plate waveguide.

Let us start with the spherical wave front *AB* radiating from the primary source *o*, as shown in Fig. 1(a). The curvature of *AB* should be a part of a circle, and we choose a quadrant in this paper with the aperture of ten wavelengths at the working frequency of 15 GHz. In order to collimate the primary radiations, we can either compress the convex air region of *ACDB* towards the source to propose a flat lens (FL) of *A’C’D’B’*, or alternatively, squeeze the rectangular air space of *A’M’N’B’* outwards to create a plano-concave lens (PL) of *AMNB*. Both of the schemes will make it equal of the travelling light path from the primary source to the outer boundaries of the transformation-optical lenses, and thus convert the spherical wave to the plane wave using the prescribed gradient-index transformational media. However, the flat lens only deals with the internal space between the source and the spherical wave front representing the most straightforward design to flatten the primary radiation directly, which is therefore much more suitable for the design of compact lens system. On the other hand, the plano-concave lens actually offers an additional cover to collimate the spherical wave front. The travelling distance from the source to the plane wave front out of the PL would be larger than the FL design, with an additional travelling space of *AMNB* acting as the collimating lens in a transformational way.

**Figure 2:**
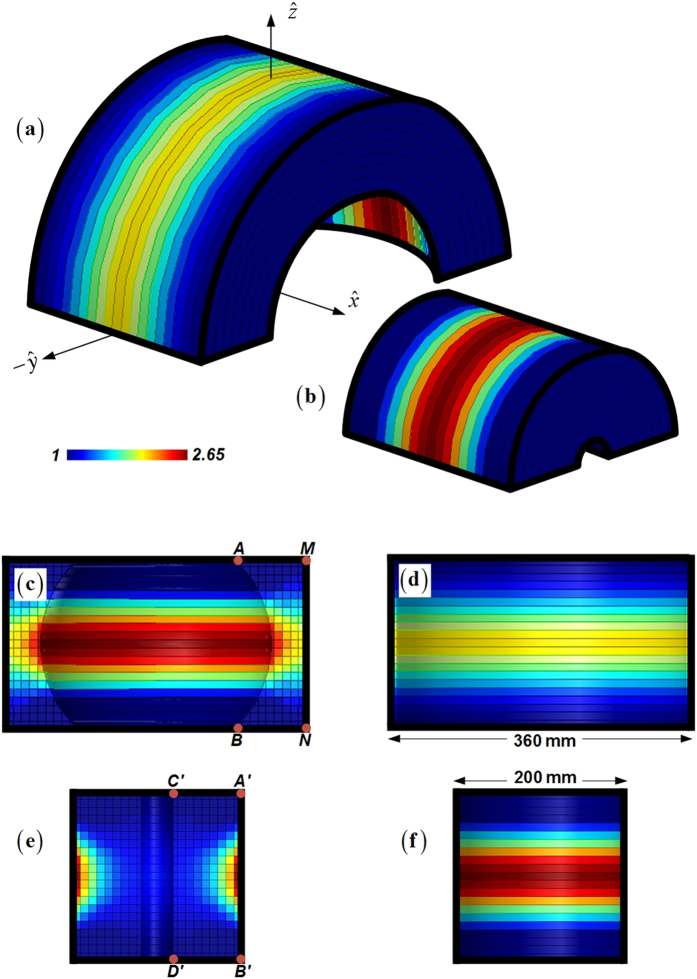
Barrel-vault constructions of the 3D fan-beam lenses. (**a**) 3D view of the RPL. (**b**) 3D view of the RFL. (**c**) Bottom view of the RPL. (**d**) Top view of the RPL. (**e**) Bottom view of the RFL. (**f**) Top view of the RFL.

According to the quasi-conformal transformation optics (QCTO)^17^, the permittivity tensors between the distorted and rectangular mapping spaces are calculated through 
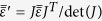
 where *J* is the Jacobian transformation matrix between the local quasi-orthogonal and Cartesian coordinates^26^. Due to the comparative conformal module of the two conversions, the value ranges of the transformed materials are roughly the same. As shown in Fig. 1(a), the PL has the minimum and maximum relative permittivity of 0.38 and 2.56, while the FL as shown in Fig. 1(b) has the minimum and maximum relative permittivity of 0.27 and 2.65. After replacing the epsilon-less-one materials by the air to remove the bandwidth limitation in the practical implementation, we properly sample the fine permittivity map to have fewer compositions^27^, as shown in Fig. 1(c,d). Every intact unit has the size of 10 mm × 10 mm, and the exterior layer has 5 mm for the thickness. In the next step, we embed both of the 2D transformation-optical lenses into the parallel plate waveguide for the purpose of fan-beam synthesis. The parallel plate waveguides and both of the lenses are chosen to have the same height of *h* = 10 mm, and the same width of *w* = 200 mm as the lens aperture. Due to the different focal lengths, the parallel plate waveguides we employed to embrace the PL and the FL would have different lengths with *l*_*PL*_ = 180 mm and *l*_*FL*_ = 100 mm, as shown in Fig. 1(e,f). In order to generate the 3D barrel-vault lenses, we revolve both of the 2D structures in Fig. 1(c,d) around the focal point along the *x*-axis 90 degrees in ±*y* direction respectively. The rotation makes these blocks become arch pillars, as shown in Fig. 2, with the outer profiles of both lenses being cylindrical but having different radius. The rotated plano-concave lens (RPL) has larger interior shaping of a beer barrel, and the inner air space of the rotated flat lens (RFL) forms an oil barrel. The RPL owns the close-to-one dielectrics in the exterior layer and larger permittivity materials in the innermost layer. In contrast, the exterior layer of the RFL has larger permittivity values and the innermost layer possesses the dielectrics close to air.

**Figure 3:**
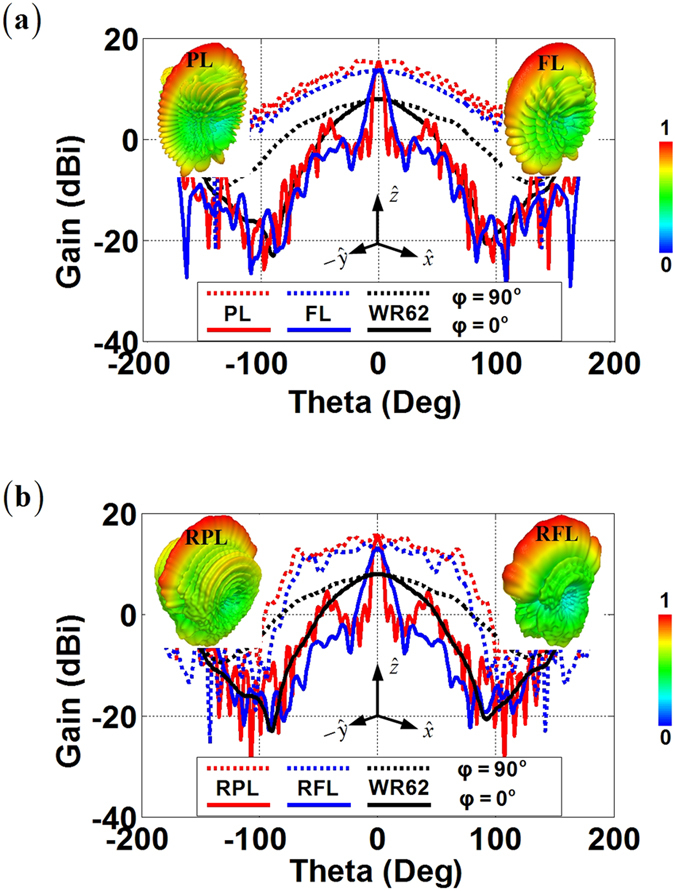
Radiation performances of the transformation-optical fan-beam lenses at 15 GHz. (**a**) 2D fan-beam lenses. Comparison is demonstrated for the far-field radiations of the PL, FL, and bare WR62 feeding waveguide. (**b**) 3D fan-beam lenses. Comparison is demonstrated for the far-field radiations of the RPL, RFL, and bare WR62 feeding waveguide.

A full-wave finite-element simulation (Ansoft HFSS v15) is then performed to test our proposed design. When we place the WR62 standard waveguide on the focal point of the PL, the FL, the RPL, and the RFL, all of the four lenses obtain very nice fan-beam radiations at 15 GHz as shown in Fig. 3. The PL and the FL have the 3-dB beamwidths of 6.94 degrees and 12.26 degrees respectively in the *xz*-plane, and 89.84 degrees and 87.76 degrees respectively in the *yz*-plane, with the maximum gain of 15.20 dBi and 13.68 dBi respectively. Such results are reasonable since the primary radiation from the feed is only collimated in the horizontal plane where the PL and the FL locate and have greatly improved the radiation from the WR62 feeding waveguide with only 7.97 dBi gain. On the other hand, the RPL and the RFL have the 3-dB beamwidths of 6.92 degrees and 13.58 degrees respectively in the *xz*-plane, and 92.14 degrees and 87.02 degrees respectively in the *yz*-plane. The RPL and the RFL also achieve well collimated beams with the maximum gain of 15.55 dBi and 13.01 dBi respectively. In the meanwhile, both of the 3D barrel-vault lenses do not spatially collimate the radiation in the unwanted dimensions. This is quite different from the TPR which also collimates the primary energy by the truncated apertures. The ray emergence angle out the lenses roughly maintains the same of the incidence, and the beamwidth in such dimensions experiences no sudden shrink from the feeding waveguide having the 3-dB beamwidths of 89.70 degrees in the *yz*-plane. Therefore, such 3D barrel-vault lenses should offer the immediate benefits in the fan-beam synthesis which can obtain highest possible gain in the desired dimension, while keeping nearly the same wide angular beamwidth as the primary source in the other dimension.

**Figure 4:**
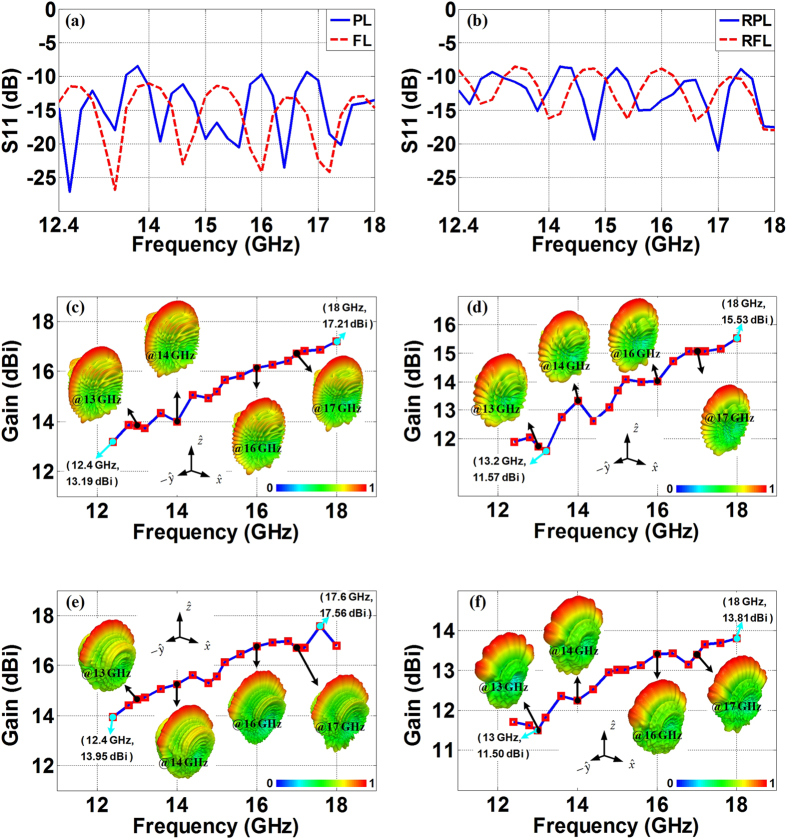
Radiation performances of the transformation-optical fan-beam lenses from 12.4 GHz to 18 GHz. The reflection coefficients of the 2D fan-beam lenses (**a**), and the 3D fan-beam lenses (**b**), The gains of the PL (**c**), the FL (**d**), the RPL (**e**), and the RFL (**f**).

Figure 4 demonstrates the bandwidth properties of these designs and their far-field radiation patterns throughout the Ku-band. We can observe that all the lenses within this frequency range have satisfactory reflection coefficients around −10 dB as shown in Fig. 4(a,b), and the 3D lenses working in the open space do not degrade much for the impedance matching compared with the 2D lenses embedded in the parallel plate waveguides. At the same time, both of the 2D and the 3D lenses function smoothly in a wide frequency range. As we expected, the PL/RPL and the FL/RFL achieve considerably nice fan-beams from 12.4 GHz to 18 GHz. The radiations for all these fan-beam lenses become stronger when the frequency goes up due to the fact that the aperture of the lenses becomes electrically larger, and this is represented by the gradually rising gains and narrowed beamwidths as demonstrated in Fig. 4 and in Table 1.

**Figure 5:**
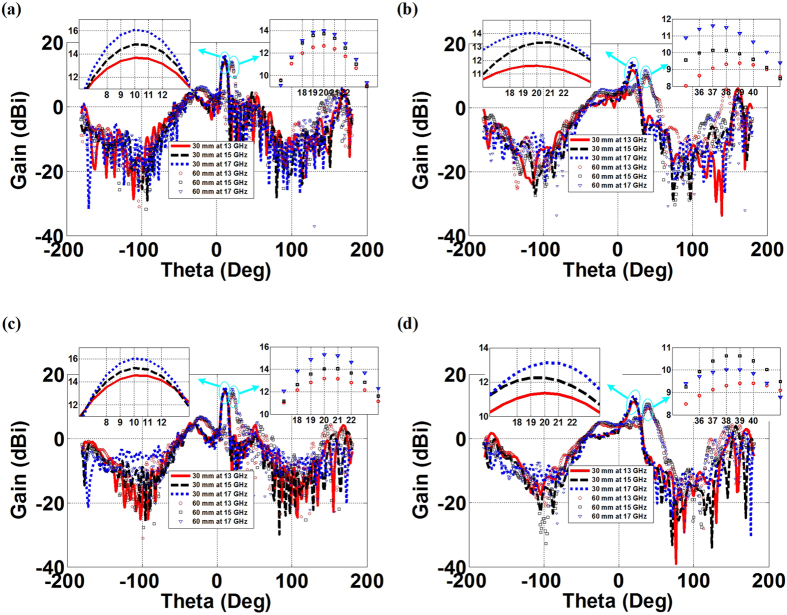
Beam steering properties of the transformation-optical fan-beam lenses with 30 mm and 60 mm off-axis feeding. (**a**) The PL, (**b**) The FL, (**c**) The RPL, and (**d**) The RFL.

The beam steering ability of the proposed fan-beam lenses are then tested in Fig. 5 with a lateral shift of the WR62 feeding waveguide within the lens system. We can observe that by moving the feed away in the *-x*-direction from the focal point to have off-axis feeding will consequently lead to a change of the main beam direction. As demonstrated, every 30 mm displacement approximately corresponds to a 10-degree and 19-degree steering angle of the fan-beam radiations, respectively, for the PL(RPL), and the FL(RFL) at 15 GHz, as shown in Fig. 5. In the meanwhile, we find that such scanning abilities of the fan-beam lenses have the merit of frequency dispersionless, and the steering angle of the main beams from the PL(RPL), and the FL(RFL) scarcely varies within the demonstration frequency range of the Ku-band for the same displacement of the feed. For example, at 13 GHz, 15 GHz, and 17 GHz, 30 mm off-axis feeding will have the same resulting scanning emissions for each demonstration of the PL and RPL having 10-degree steering main lobes, and the FL and the RFL having 19-degree steering main lobes, and this is also valid for 60 mm off-axis feeding for the proposed 2D and 3D fan-beam lenses as tested in Fig. 5.

**Figure 6:**
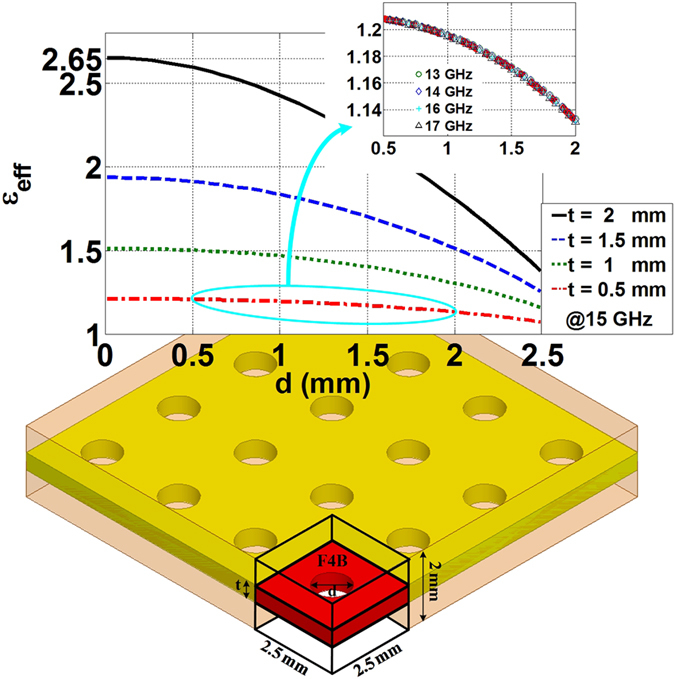
Dielectric synthesis of the proposed fan-beam lenses based on the effective media theory through perforated F4B dielectrics. The unit cell has the dimension of 2.5 × 2.5 × 2 mm^3^, *t* refers to the thickness of the employed F4B layer, *d* refers to the diameter of the drilled holes.

Finally, we propose to construct these four types of fan-beam lenses through multiple-layered F4B dielectrics with non-uniformed perforations. In the view of the effective media, such a design can be considered as a homogeneous material replacing the air and the F4B sheets, where the volume ratio between the two mixed dielectrics would consequently determine the permittivity value^28^. We can observe in Fig. 6 that the effective permittivity achieves the value ranges of [1.38, 2.65], [1.26, 1.93], [1.16, 1.51], and [1.07, 1.21] respectively at 15 GHz, with the host F4B thickness of 2 mm, 1.5 mm, 1 mm, and 0.5 mm within the periodic unit cell of 2.5 × 2.5 × 2 mm^3^ where the hole diameter varies from 0 to 2.5 mm. Such a non-resonant method of dielectric mixing of F4B and air holes is also frequency dispersionless, and this is reflected by the nearly overlapped curves from the parameter retrieval at different frequencies in Fig. 6. However, the diameter of the holes cannot be chosen as large as 2.5 mm in the real fabrication and the separation distance between two perforations should be large enough to sustain the integration of the host dielectrics, otherwise a small machining error would possibly lead to the F4B layer breaking into pieces while performing the practical implementation.

**Figure 7:**
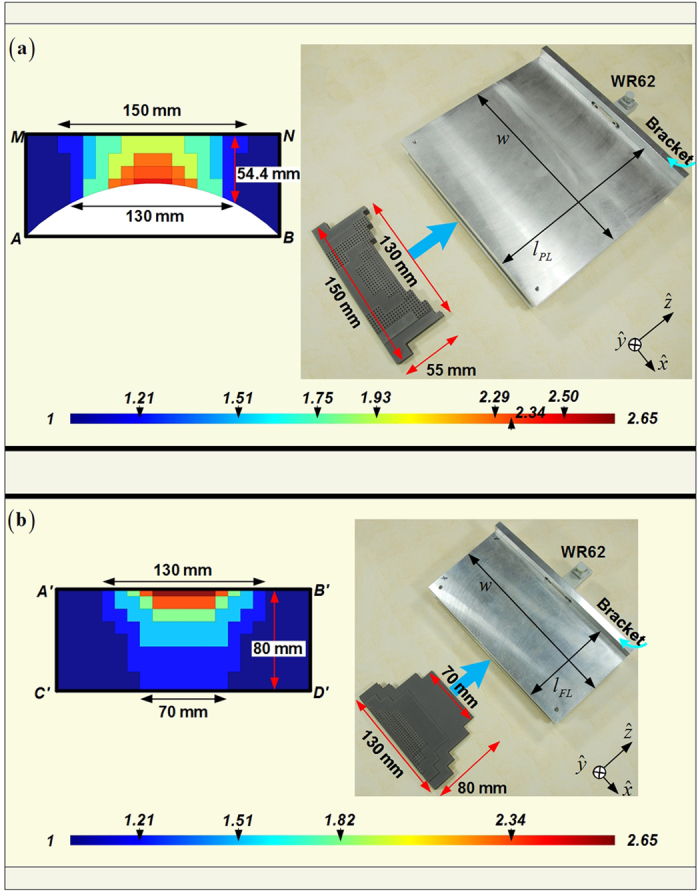
Concise version of 2D fan-beam lenses, as well as their manufactured photos. (**a**) The PL, (**b**) The FL. Both lenses would be embedded in the parallel plate waveguide as in Fig. 1(e,f) while they are functioning. The supporting bracket has the size of 200 × 50 × 8 mm^3^ for both of the lenses. The parallel plate waveguide and the bracket are both made of aluminum in the fabrication.

**Figure 8:**
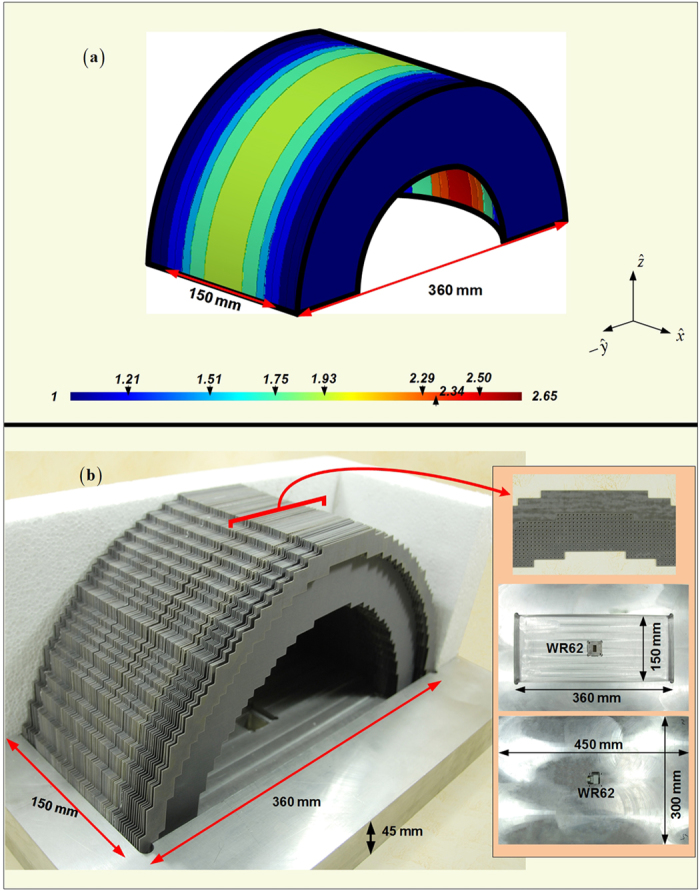
Concise version of the RPL (a)and its manufactured photo (b) on the lens supporting bracket. The embedded pictures in (**b**) refer to the center part of the middle 4-layer (2mm) of the perforated F4B dielectrics, and the supporting bracket with WR62 feed.

**Figure 9:**
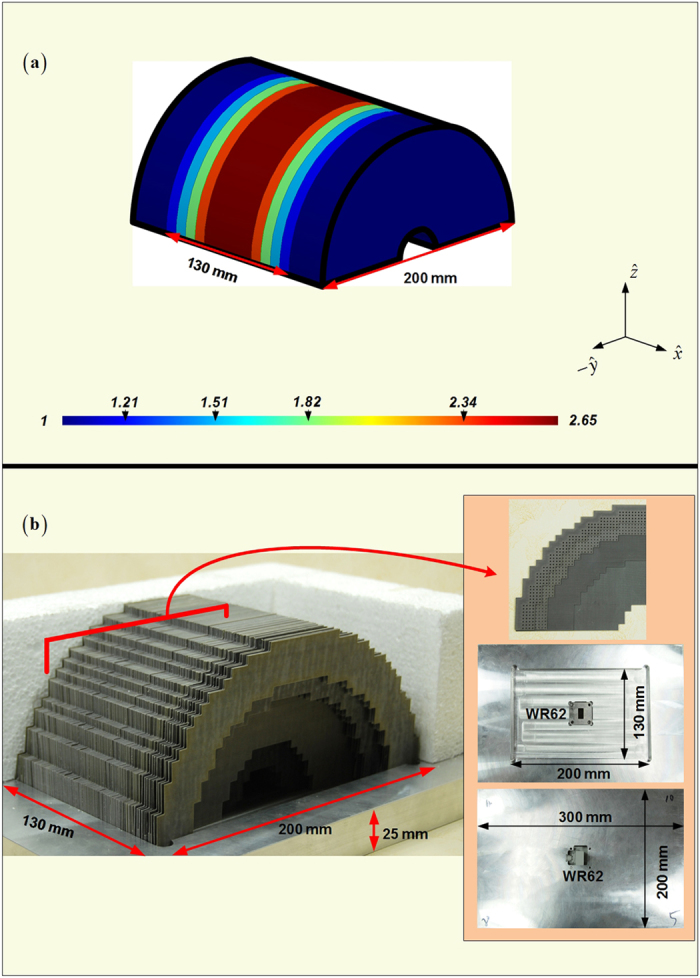
Concise version of the RFL (a)and its manufactured photo (b) on the lens supporting bracket. The embedded pictures in (**b**) refer to one half of the middle 4-layer (2mm) of the perforated F4B dielectrics, and the supporting bracket with WR62 feed.

With the aim to achieve the balance between the easier fabrication and the satisfactory radiation, we continue to optimize gradient compositions of the four fan-beam lenses. Because the curvature *AB* we employed in Fig. 1(a) for the transformation design is a part of the circle, the conformal module of the distorted coordinates in every radial zone around the circle center are roughly the same when transformed into or converted from the standard square in the Cartesian coordinate system. This is reflected by the similar-color dielectric blocks of nearly the same permittivity values within the PL and FL as shown in Fig. 1. Therefore, we are having the opportunity to conduct the further approximation and use just several effective media to replace the original transformation lens designs, and this would no doubt offer the convenience in the fabrication. Figures 7, 8 and 9 demonstrate the concise version of the four lenses and their practical build-up with layered F4B dielectrics (relative permittivity 2.65; loss tangent 0.001). As is shown, the PL and the FL are replaced by 7 and 5 stair-stepping dielectric bars, with their 3D counterparts of the RPL and the RFL barrel-vaults simplified accordingly. In such a way, only ten different values of the effective media are required in these final designs. Moreover, just five permittivity values need the perforation, and the other five relative permittivity values of 1, 1.21, 1.51, 1.93, 2.65 can directly employ the air or the available thickness of F4B layer with no perforation. In addition, both of the PL and the FL can be simply constructed by only using layers of the 0.5 mm thick F4B with non-uniformed perforations in the fabrication. For example, if the compositions of the transformational lenses require 1.5 mm thick F4B dielectrics according to the parameter retrieval in Fig. 6, we could use three layers of 0.5 mm thick F4B instead. When it comes to the 3D fan-beam lenses of the RPL and the RFL, we do not follow the same procedure as the transformational designs from Figs 1 and 2 by rotating the 2D fan-beam lenses 90 degrees directly in ±*y* directions respectively for their 3D versions to generate the fan-beams, since such a rotation will turn the perforations into the hollow tubes in the barrel-vaults, which will make the 3D lenses even more complicated and difficulty to implement. On the other hand, both of the RPL and the RFL have gradient-index dielectric layers parallel to the *yz*-plane as shown in Figs 8(a) and 9(a), and therefore, we can employ the non-uniformed perforated dielectrics to represent all these layers of the 3D barrel-vaults, as demonstrated in Figs 8(b) and 9(b), which is the same method as we construct the 2D fan-beam lenses.

**Figure 10:**
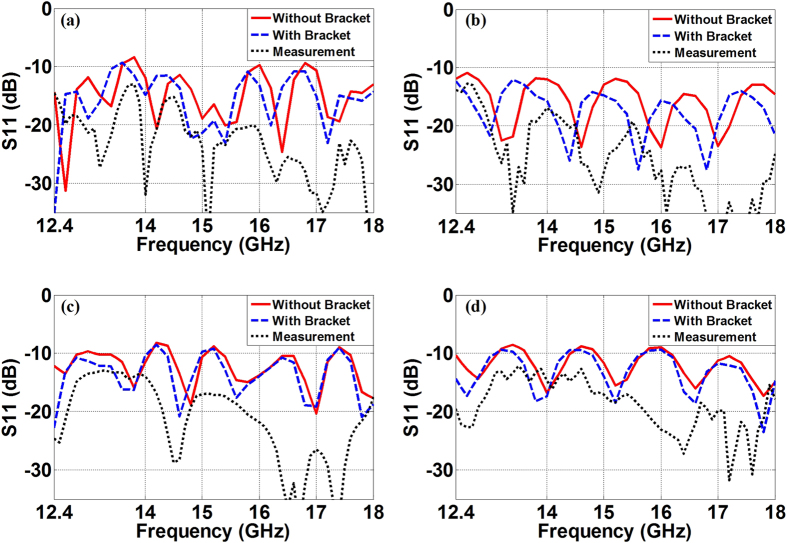
Reflection coefficients of the proposed fan-beam lenses throughout the Ku-band with simulation results for the concise fan-beam lenses with/without supporting brackets and measured results for the corresponding manufactured designs. (**a**) The PL, (**b**) The FL, (**c**) The RPL, and (**d**) The RFL.

**Figure 11:**
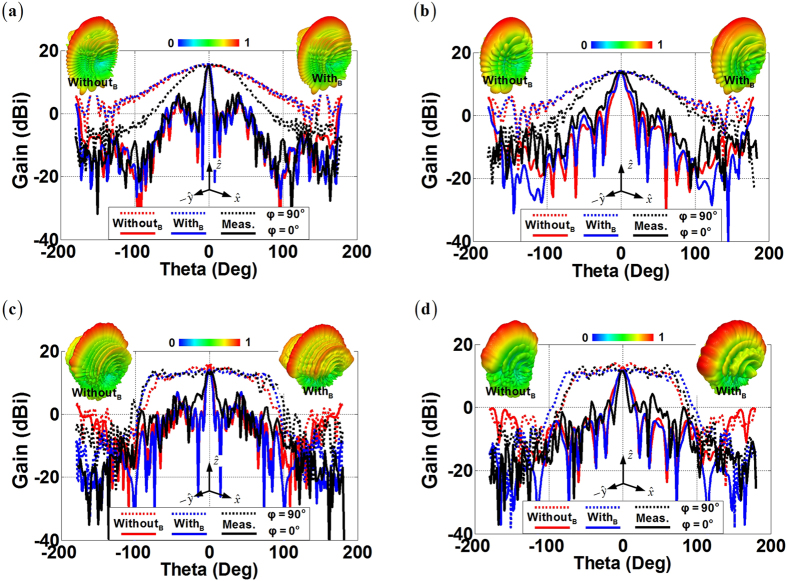
Comparisons of far-field radiation patterns of the proposed fan-beam lenses at 15 GHz with simulated gains of concise lenses with/without supporting brackets, and the measured gains of the corresponding manufactured designs. The embedded 3D radiation patterns refer to the simulated results of the concise lenses with/without supporting brackets. (**a**) The PL, (**b**) The FL, (**c**) The RPL, and (**d**) The RFL.

Experimental tests are then carried out to verify the proposed F4B lenses at Ku-band. As can be observed in Fig. 10, all the lenses within Ku-band have acceptable reflection coefficients around −10 dB from the simulations. The adding of the supporting bracket for the practical implementation does not cause additional reflections for the four fan-beam lenses, but only a little bit of the migration of the frequency response. The experimental results have the same trend with simulation curves and the measured reflection coefficients are all less than −10 dB from 12.4 GHz to 18 GHz. Simulated curves from 2D and 3D lenses experience 5 fluctuations throughout the whole Ku-band with all the measured results basically accordant to the simulations. However, the consistence between results from the simulations and the measurements shows that data in 2D cases agree better than those in 3D cases. In addition, we can also observe that the measured reflection coefficients are roughly 4 dB less than the simulation ones, and such reduced reflections become more obvious in the higher frequency. These differences mainly ascribes to the ohmic losses in the fan-beam lenses from aluminum and F4B dielectrics during the practical implementation, while in the simulation we assume the parallel plate waveguide, the supporting bracket, and the dielectric compositions of the fan-beam lenses are perfectly lossless. In the transmitting concerning the radiation patterns, we can observe in Fig. 11 that both of the 2D and the 3D perforated F4B lenses and their concise transformation prototypes with and without the supporting bracket are capable of achieving satisfactory fan-beam radiations, and the measurement results match considerably well with the far-fields from the full-wave simulations. Table 2 further compares the radiation properties of the proposed fan-beam lenses at 15 GHz of the original simplified version in Figs 1 and 2, the concise version with/without the supporting brackets, and the manufactured ones. We can observe that the concise lenses generate nearly the same quality of the fan-beams as we devised in the original transformation designs. Adding of the supporting bracket does not degrade the radiation performance, and especially it greatly enlarges the beamwidth in the *yz*-plane for the 3D fan-beam lenses. The experimental results show that our manufactured fan-beam lenses satisfactorily maintain the original transformation design for the fan-beam synthesis. The manufactured PL and FL have the 3-dB beamwidths of 8.84 degrees and 18.62 degrees respectively in the *xz*-plane, and 60 degrees and 74.88 degrees respectively in the *yz*-plane, with the measured gains of 15.23 dBi and 13.49 dBi respectively from the measurements. In the meanwhile, the manufactured RPL and RFL have the 3-dB beamwidths of 11.2 degrees and 9.94 degrees respectively in the *xz*-plane, and 170.9 degrees and 138.26 degrees respectively in the *yz*-plane from the measurements, with the measured gains of 12.68 dBi and 11.61 dBi respectively. These measurement results further verify our designs of the fan-beam generators in both 2D and 3D cases for the successfully synthesizing the directive beam in the desired dimension, while maintaining the wide beamwidth in the other dimension.

## Discussion

We have demonstrated transformation-optical fan-beam synthesis by proposing four kinds of gradient-index lenses. All of our devices have been proved to be efficiency in obtaining fan-beams over a broad frequency range while possessing the great ability of the wide angular scanning. The 3D barrel-vault lenses have roughly the same radiating capacities as their counterparts of the 2D lenses in the parallel plate waveguide, and the structure extension does not degrade their performance of the fan-beam synthesis. Especially, the wide beamwidth in the dimensions other than the lens locating plane has still perfectly been maintained. Therefore, both of our 2D and 3D lenses should function very well to generate the fan-beam signals. In addition, the proposed 3D lenses have much bigger perception windows compared with the parallel plate waveguide embraced PL and FL with an aperture of *A*_*PL(FL)*_ = *h* × *w* = 2 × 10^3^ mm^2^ to connect the outside. The exterior surfaces of barrel-vaults are *A*_*RPL*_ = *π* × *l*_*PL*_ × *w* = 1.13 × 10^5^ mm^2^ for the RPL and *A*_*RFL*_ = *π* × *l*_*FL*_ × *w* = 6.28 × 10^4^ mm^2^ for the RFL respectively. Such structural characteristics are attributed to the rotation of the whole antenna system of the 2D PL and the 2D FL, offering great convenience for the radar antenna system to access the outside information through just a rotation of the feed inside the 3D fan-beam barrel-vaults.

We have also proposed to utilize the non-uniformed perforation F4B dielectrics to construct all of these fan-beam lenses and experimentally verified their excellence in the fan-beam generation. The concise design of fan-beam lenses for the fabrication should be also valid in other transformation-optical designs, and can be further explored for other useful applications. The physics behind is the generation of quasi-identically conformal modules in the distorted coordinates so that we are able to perform the further simplification accordingly. This operation is different from previous designs using QCTO, where the sizes of dielectric compositions of the transformational devices are limited to the half wavelength of travelling waves^20^,^21^,^22^,^23^,^27^. In this sense, it would be possible to propose much more concise designs with fewer dielectrics through manipulating the mesh generation concerning the original distorted space, and facilitate the final fabrications for the transformation-optics based devices.

## Additional Information

**How to cite this article**: Yang, R. *et al*. Transformation-optical Fan-beam Synthesis. *Sci. Rep.* **6**, 20530; doi: 10.1038/srep20530 (2016).

## Figures and Tables

**Table 1:** The radiation performances of fan-beam lenses throughout the Ku-band.

		PL	FL	RPL	RFL
Gains (dBi)	Ku-band	[13.19, 17.21]	[11.57, 15.53]	[13.95, 17.56]	[11.50, 13.81]
Beamwidths (Degrees) *xz*-plane/*yz*-plane	13 GHz	7.72/101.10	12.92/97.92	8.00/93.44	14.20/87.18
	14 GHz	7.04/94.97	12.10/86.50	7.48/89.62	13.58/87.02
	16 GHz	6.38/82.20	12.14/83.60	6.60/73.68	13.02/88.54
	17 GHz	6.08/77.16	11.38/73.44	6.14/72.86	12.86/92.90

**Table 2:** Comparisons of the proposed fan-beam lenses at 15 GHz of the original simplified version, the concise version with/without supporting brackets, and the manufactured version.

		PL	FL	RPL	RFL
Gains (dBi)	Simplified Lenses	15.20	13.68	15.55	13.01
	Concise Lenses (without_B & with_B)	15.13	13.95	15.59	12.75
		15.68	14.09	14.19	11.71
	Measurements	15.23	13.49	12.68	11.61
Beamwidths (Degrees)	Simplified Lenses	6.94/89.84	12.26/87.76	6.92/92.14	13.58/87.02
	Concise Lenses (without_B & with_B)	6.94/90.34	11.02/81.04	7.02/76	12.04/93.96
		6.7/89.84	9.42/79.8	6.56/165.44	15.39/120.96
	Measurements	8.84/60	18.62/74.88	11.2/170.9	9.94/138.26

The bandwidth properties are represented in the *xz*-plane/*yz*-plane for the 2D and the 3D lenses.
